# Design of Bi-Material Triangle Curved Beam Honeycomb Metamaterial with Tunable Poisson’s Ratio, Thermal Expansion, and Band Gap Characteristics

**DOI:** 10.3390/ma18102408

**Published:** 2025-05-21

**Authors:** Zelong Wang, Yong Cheng, Huichuan Zhao, Han Zhang

**Affiliations:** 1 Hebei Key Laboratory of Mechanical Reliability for Heavy Equipments and Large Structures, Yanshan University, Qinhuangdao 066004, China; wangzelong@stumail.ysu.edu.cn; 2 Science and Technology Quality Department Innovation Center, China 22MCC Group Co., Ltd., No. 16 Xingfu Road, Fengrun District, Tangshan 063205, China; zhao.yunqiong@163.com; 3 Institute of Acoustics, Chinese Academy of Sciences, Beijing 100190, China

**Keywords:** metamaterials, adjustable Poisson ratio, tunable thermal expansion, band structure

## Abstract

Metamaterials, owing to their exceptional properties such as a negative Poisson’s ratio, phonon band gap, and energy absorption, have garnered significant interest in aerospace, automotive transportation, and other domains. The increasing demand for metamaterial structures with diverse specialized attributes requires innovative design approaches. In this study, a novel bi-material triangular curved beam honeycomb metamaterial (BTBM) is designed, which exhibits a tunable Poisson’s ratio (PR), coefficient of thermal expansion (CTE), and band gap characteristics. These properties are intrinsically coupled through the geometric and material design of the bi-material triangular curved beam structure, meaning that adjustments to the unit cell configuration simultaneously influence PR, CTE, and band gap behavior. This dual-mode control offers versatile design strategies for multifunctional metamaterials. The energy band structure is calculated using finite element simulation analysis, and its accuracy is validated by computing the transmission characteristic curve. Numerical simulations were performed to systematically analyze the coupled effects of geometric parameters and material combinations on the PR and CTE. The results demonstrate significant tunability of these mechanical properties through parametric optimization. The results of this study provide valuable insights into the design and optimization of metamaterial structures with tailored properties for various applications.

## 1. Introduction

With the continued advancements in material science research, mechanical metamaterials [1,2,3,4] have garnered significant interest due to their remarkable mechanical properties and vast potential for applications. Mechanical metamaterials, which are artificially designed composite structures, exhibit unique mechanical behaviors that are unparalleled in natural materials. These include, but are not limited to, negative Poisson’s ratio (NPR) [5,6,7], negative thermal expansion (NTE) [8,9,10], energy absorption capabilities [11,12], adjustable band gaps [13,14,15,16], and exceptional vibration isolation properties. These exceptional properties hold immense promise for a wide range of applications. Notably, NPR metamaterials have shown particular promise in acoustic and multiphysics applications [17,18,19]. For instance, by fine-tuning the Poisson’s ratio (PR), the compressive, tensile, and impact resistance of the material can be optimized, making it suitable for engineering applications under extreme conditions. Additionally, the adjustable thermal expansion (CTE) ensures consistent accuracy and performance of equipment across varying temperatures, especially crucial in high-precision equipment and optical instruments. Furthermore, by designing materials with specific phonon band gaps, the propagation of sound waves can be precisely controlled, enabling noise reduction, sound insulation, and even acoustic stealth. As research progresses, the applications of mechanical metamaterials are expected to expand further, revolutionizing various fields.

Metamaterials, through their intricately designed microstructures, such as honeycomb [20,21] and origami patterns [22], have the remarkable ability to modulate PR from positive to negative domains. This versatile feature empowers metamaterials to dynamically adjust the interplay between transverse and longitudinal strains in response to external stimuli, underpinning their exceptional performance in shock mitigation, energy absorption, and shape memory functionalities. For instance, Fu et al. [23] innovatively devised a three-dimensional chiral honeycomb structure, which exhibited an NTE effect, signifying a significant advancement in the field. Furthermore, the pursuit of the NTE effects has also garnered significant attention, with specific material designs facilitating this phenomenon. Notably, Miller et al. [24] introduced a triangular configuration where temperature variations trigger differential expansions among its constituent elements, thereby tuning the CTE. This underscores the potential for designing metamaterials that can concurrently manipulate both the PR and the CTE properties, representing an emerging trend in materials science. In this context, Wei et al. [25] successfully demonstrated a cellular structure capable of simultaneous regulation of these two crucial parameters. Moreover, the ubiquitous presence of detrimental vibrations and noise in aerospace, transportation, and myriad other industries poses significant challenges to the reliability and operational precision of machinery and equipment. Consequently, the development of band gap structures that effectively isolate sound and dampen vibrations [26,27,28] has garnered considerable research interest. Liu et al. [29] made a noteworthy contribution by designing a composite structure that not only allowed the tuning of the PR and the CTE, but also exhibited desirable band gap characteristics, offering a multifaceted solution to address these challenges. In summary, the ongoing research in metamaterials, particularly those focusing on the concurrent manipulation of the PR, the CTE, and band gap properties [30,31,32], holds immense promise for advancing various technological sectors and enhancing the performance of modern systems.

In this paper, we introduce a novel bi-material triangular curved beam honeycomb metamaterial (BTBM), conceived through the innovative integration of a bi-material triangle with a curved beam honeycomb configuration. This design aims to endow the structure with tunable properties, specifically, its CTE, PR, and bandgap characteristics. Leveraging the Bloch theorem as the theoretical framework, we employ finite element analysis to meticulously compute the band structure of the proposed structure. Furthermore, we validate the accuracy of our band structure calculations by analyzing the transmission characteristic curves, thereby ensuring the robustness of our methodology.

To gain insights into the mechanical behavior of this intricate design, we conduct a systematic investigation into the influence of geometric parameters and material pairings. Our findings reveal that through judicious material selection and shape optimization, this structure exhibits remarkable capabilities in achieving coupled modulation of both the PR and the CTE, accompanied by a substantial total effective bandgap width. This unique combination of properties underscores the potential of our bi-material triangular curved beam for applications requiring tailored thermal and mechanical responses.

## 2. Structural Design and Method

In this paper, a kind of bi-material triangle curved beam honeycomb metamaterial (BTBM) is proposed by combining the bi-material triangle and curved beam honeycomb. The effective PR and CTE of the BTBM are calculated by numerical simulation. The band structure was numerically determined using Bloch’s theorem, with its accuracy validated through transmission spectrum analysis.

### 2.1. Design of Structure

**Figure 1:**
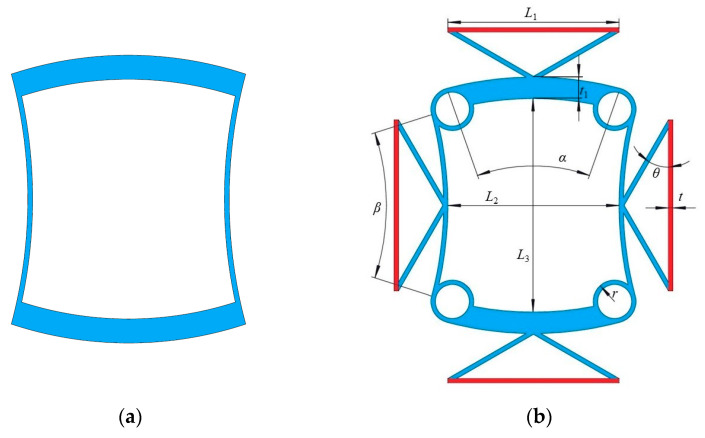
BTBM design strategy and geometry. (**a**) Negative Poisson’s ratio material composed of two concave curved beams and two convex curved beams; (**b**) unit cell of BTBM (red represents material 1 and blue represents material 2).

The NTE phenomenon observed in the bi-material model is primarily attributed to the dissimilarity in the CTE between the two constituent materials. As the temperature varies, the disparity in CTEs results in the characteristic ‘heat shrinkage and cold expansion’ behavior within a defined temperature range. Specifically, upon an increase in temperature, the volume of the structure contracts, whereas a decrease in temperature leads to its expansion. Efficient realization of NTE is achieved through the design of a bi-material triangular structure. This approach harnesses the contrasting CTEs of the two materials, giving rise to the desired thermal expansion behavior. Negative Poisson’s ratio materials are realized by using a material composed of two concave curved beams and two convex curved beams, as shown in Figure 1a, where the thickness of the concave curved beam is greater than that of the convex curved beam. When the material is subjected to compressive force in the horizontal direction, the convex curved beam deforms inward, which in turn causes the concave curved beam to deform inward, thereby achieving the negative Poisson’s ratio effect. To reduce stress concentration and manufacturing complexity, circular rings are used to connect two adjacent curved beams. Furthermore, a bi-material triangular configuration is connected to the outer sides of the four curved beams, thereby realizing the BTBM design, as shown in Figure 1b. The geometric parameters of the BTBM include the bottom edge length *L*_1_ and angle *θ* of the triangle, the angles *α* and *β* of the curved beams, the inner distances *L*_2_ and *L*_3_, the radius *r* of the circular rings, the thickness *t*_1_ of the concave curved beam, and the thickness *t* of the remaining parts of the material. Red represents material 1 with a thermal expansion coefficient of *α*_1_, and blue represents material 2 with a thermal expansion coefficient of *α*_2_.

Three representative materials—Al, Steel, and Invar alloy—were systematically investigated to evaluate their combinatorial effects on the structural mechanical performance. The specific performance parameters of the three materials are shown in Table 1.

### 2.2. Numerical Simulation

In order to better study the Poisson‘s ratio, thermal expansion, and band gap characteristics of the structure, the materials are selected for the model are Al, Steel and Invar. The single cell structure size of the BTBM is set as *L*_1_ = 16 mm, *θ* = 30°, *α* = *β* = 30°, *t* = 0.4 mm, *t*_1_ = 2 mm, and *r* = 2 mm.

**Figure 2:**
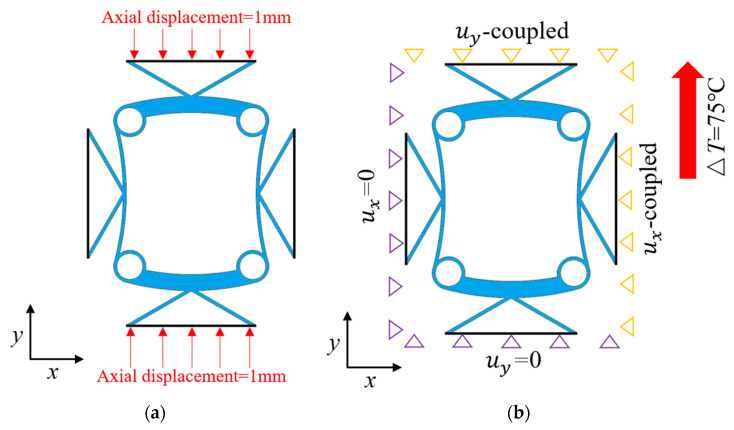
(**a**) The PR constraints; (**b**) the CTE constraints and temperature changes.

As shown in Figure 2a, the upper boundary of the BTBM structure was constrained to a displacement of 1 mm negative to the *y*-axis, and the lower boundary was constrained to a displacement of 1 mm positive to the *y*-axis. The left and right boundaries were not constrained. The transverse and longitudinal deformations of the BTBM were determined by finite element analysis, so as to realize the calculation of the effective PR.

In the context of finite element analysis, as depicted in Figure 2b, the left boundary of the structure was subjected to a displacement constraint of (*x* = 0), while the lower boundary was constrained to (*y* = 0). The upper and right boundaries remained as free constraints, permitting unrestricted movement. The temperature was gradually increased from 25 °C to 100 °C (Δ*T* = 75 °C), and the resulting displacement data (Δ*D*) were recorded. Following the method of Wei et al. [25], the effective coefficient of thermal expansion (CTE) for a unit cell with initial characteristic length (*D*) was calculated as:

(1) $$\alpha \;=\frac{\Delta D}{D\cdot \Delta T}$$

**Figure 3:**
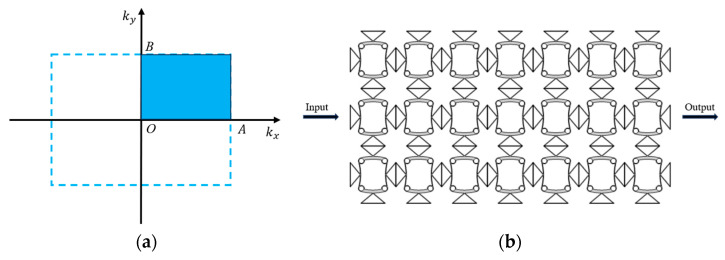
(**a**) The first Brillouin region and irreducible Brillouin region (shaded in blue); (**b**) the 7 × 3 lattice array of structures.

To simulate the wave propagation characteristics in an ideal phononic crystal, periodic boundary conditions were applied to all model boundaries using Floquet–Bloch theorem implementation. These conditions ensured the periodic propagation of elastic waves within the periodic structure. The dispersion relations were determined through systematic scanning of wave vectors (*k*) along the path B-O-A in the irreducible Brillouin zone, capturing the complete directional dependence of wave propagation characteristics. Based on the Bloch theorem, the relationship between the wave vector and frequency at the boundaries of the Brillouin zone was solved, deriving the energy band structure. Figure 3a illustrates the first Brillouin zone with its irreducible wedge highlighted (blue shaded region), corresponding to the primitive unit cell’s reciprocal space representation.

The transmission spectrum was numerically computed to verify the bandgap properties predicted by the dispersion analysis. As illustrated in Figure 3b, a 7 × 3 array configuration was utilized for this purpose. A pre-defined 1 mm displacement excitation was applied to the left boundary, and the displacement response after transmission through the structure was recorded at the right boundary. These data were then used to obtain the lateral transmission characteristic curve. To analyze the transmission characteristics in phononic crystals, a preset 1 mm displacement excitation was applied to the lower boundary. The longitudinal wave transmission characteristics of the 7 × 3 lattice metamaterial were investigated through controlled displacement excitation at the upper boundary, with the attenuated output response measured after propagation. The transmission coefficient was calculated using:

(2) $$T(\mathrm{dB})\;=\;20\log_{10}\left(\frac{A_{\mathrm{output}}}{A_{\mathrm{input}}}\right)$$

where *A*_input_ and *A*_output_ represent the displacement amplitudes at the excitation and measurement boundaries, respectively. Structural damping effects were systematically accounted for in the transmission spectrum analysis.

**Figure 4:**
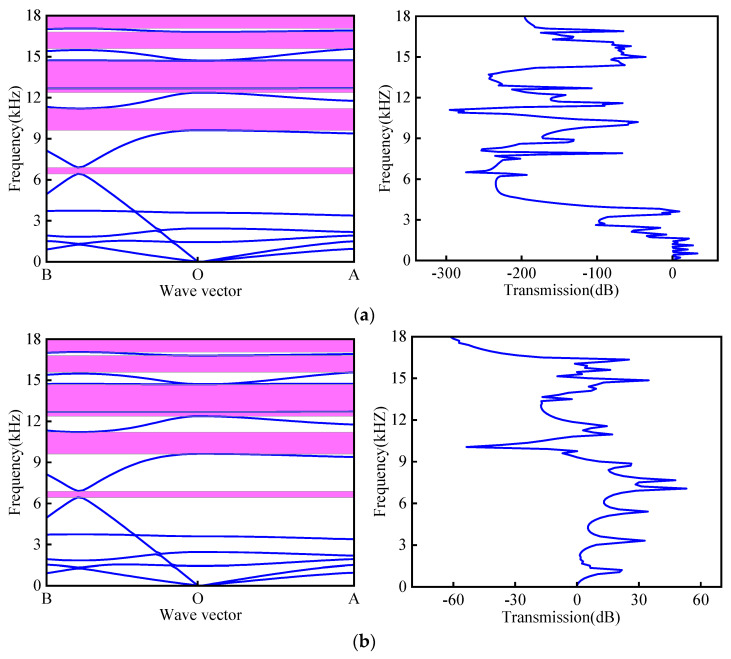
(**a**) Band structure and transverse transmission characteristic curve; (**b**) band structure and longitudinal transmission characteristic curve (Purple indicates band gap).

The calculation results are presented in Figure 4a,b. The purple region in these figures represent the band gap in a specific frequency range. Excellent agreement is observed between the attenuation bands in the transmission spectra and the bandgap regions predicted by the dispersion analysis, confirming the accuracy of the numerical calculations. Both transverse and longitudinal wave measurements exhibit significant attenuation within the theoretically predicted bandgap frequency ranges.

## 3. Results and Discussion

### 3.1. Parameter Analysis of the Effective PR

**Figure 5:**
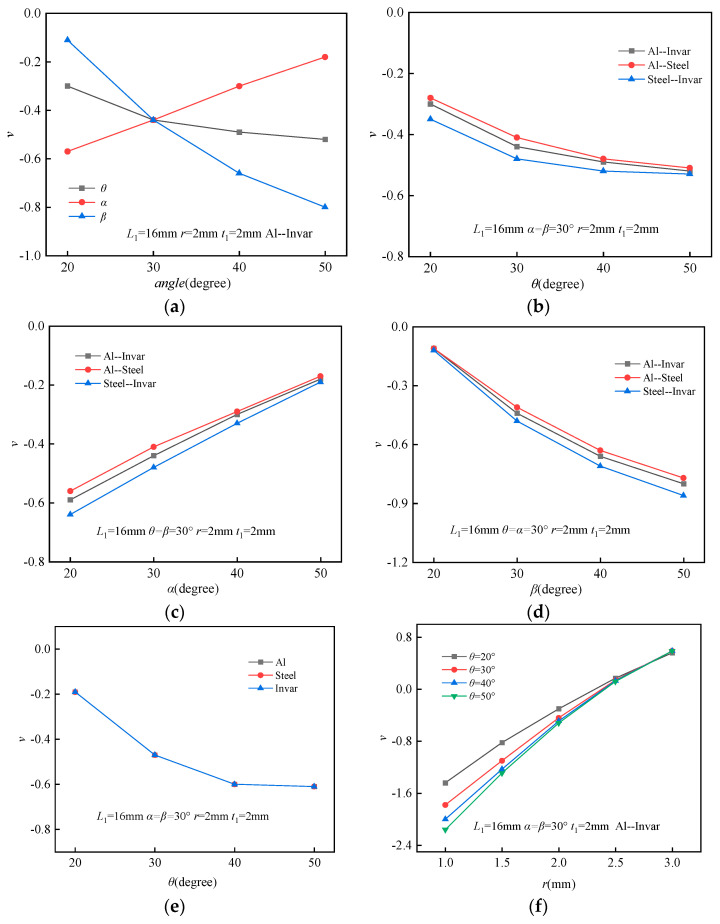

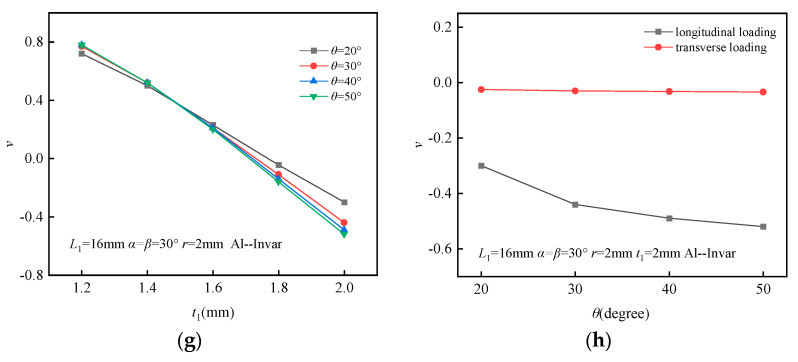
FEA of *ν* under various parametric conditions: (**a**) *θ*, *α*, *β* interactions; (**b**) *θ* variation; (**c**) *α* variation; (**d**) *β* variation; (**e**) materials variation; (**f**) *r* variation; (**g**) *t*_1_ variation; (**h**) lateral constraint case.

To systematically investigate the influence of the triangle angle (*θ*), thick beam angle (*α*), and thin beam angle (*β*) on the PR, the geometric parameters of the BTBM were fixed as follows: *L*_1_ = 16 mm, *t* = 0.4 mm, *t*_1_ = 2 mm, and *r* = 2 mm. The material used for the structure was Al–Invar. When varying a single angle (e.g., *θ*), the other two angles (*α* and *β*) were kept constant at their respective initial reference values of 30°. This approach was applied similarly when analyzing the effects of *α* and *β* individually, ensuring that the influence of each angle on the Poisson’s ratio could be independently evaluated. As illustrated in Figure 5a, the *ν* increased progressively with the enlargement of *α*. In contrast, *ν* decreased as *θ* and *β* increased, demonstrating an enhanced NPR effect. These results indicate that increasing *θ* and *β* significantly strengthens the NPR effect.

To examine the effect of the angle *θ* on the PR under varying material combinations, the geometric parameters of the BTBM were fixed at *L*_1_ = 16 mm, *α* = *β* = 30°, *t* = 0.4 mm, *t*_1_ = 2 mm, and *r* = 2 mm, while *θ* was varied at 20°, 30°, 40°, and 50°. Three material systems were systematically investigated in this work: Al–Invar, Al–Steel, and Steel–Invar bi-material combinations. The FEA results, presented in Figure 5b, reveal that the *ν* values for the three material combinations are remarkably similar, indicating that *ν* is largely independent of the material composition. As *θ* increases, *ν* gradually decreases, reaching its maximum negative value at *θ* = 50°.

To explore the influence of angles *α* and *β* on the Poisson’s ratio (PR) across various material systems, the BTBM’s geometric parameters were consistently set as *L*_1_ = 16 mm, *t* = 0.4 mm, *t*_1_ = 2 mm, and *r* = 2 mm. During the analysis of *α*, varied from 20° to 50°, *θ* and *β* were held constant at 30°. Similarly, when examining *β* within the same angular range, *θ* and *α* were fixed at 30°. The investigations utilized the same three material pairings: Al–Invar, Al–Steel, and Steel–Invar. As shown in Figure 5c,d, the Poisson’s ratio across all material combinations exhibited minimal change, reaffirming that *ν* is largely independent of material selection. The results identified two distinct trends: *ν* progressively increased with rising *α*, reaching its most negative value at *α* = 20°, while it steadily decreased with increasing *β*, maximizing the NPR effect at *β* = 50°. These observations further emphasize that although the material composition exerts negligible impact on the PR, the geometric configuration, particularly *α* and *β*, exerts significant but opposing effects on the *ν* behavior.

To further confirm the material-independence of the negative Poisson’s ratio (NPR) characteristics, supplementary simulations were carried out using single-material models. The geometric parameters of the BTBM were kept constant with *L*_1_ = 16 mm, *α* = *β* = 30°, *t* = 0.4 mm, *t*_1_ = 2 mm, and *r* = 2 mm, while systematically varying *θ* from 20° to 50°. For this purpose, three homogeneous materials—aluminum (Al), Steel, and Invar—were employed. As depicted in Figure 5e, the *ν* values for all three materials followed nearly identical trends and exhibited comparable magnitudes throughout the investigated angular range. These findings strongly support that the NPR phenomenon primarily arises from the structural geometry rather than the intrinsic properties of the materials. The uniform behavior observed across both bi-material and homogeneous systems reinforces the conclusion that geometric design, rather than material selection, governs the NPR response. Moreover, this result aligns closely with the observations reported by Liu et al. [29], who also demonstrated that Poisson’s ratio is largely insensitive to the choice of constituent materials but highly dependent on the geometrical configuration.

To investigate the influence of the ring radius (*r*) on the PR at varying angles *θ*, the geometric parameters of the BTBM were fixed at *L*_1_ = 16 mm, *α* = *β* = 30°, *t* = 0.4 mm, and *t*_1_ = 2 mm, with *θ* varied at 20°, 30°, 40°, and 50°, and *r* adjusted to 1.0 mm, 1.5 mm, 2.0 mm, 2.5 mm, and 3.0 mm. The material used for the structure was Al–Invar. The FEA results in Figure 5f show *ν* transitions from negative to positive values as *r* increases between 2.0 mm and 2.5 mm. This indicates that the positive and negative states of *ν* can be flexibly controlled by adjusting *r*, with the influence of *r* being more pronounced than that of *θ*. Furthermore, the NPR effect becomes more apparent at larger *θ* values. The *ν* values at different *θ* angles gradually converge, and the NPR effect is maximized at *θ* = 50° and *r* = 1.0 mm. Significantly, the proposed structure exhibits a much wider tunable Poisson’s ratio range (−2.2 to 0.6) compared to Liu et al.’s design (−1.0 to 0.3) [29], demonstrating superior tunability and performance.

To explore the effect of the wall thickness (*t*_1_) of the longitudinal unit on the PR at varying angles *θ*, the geometric parameters of the BTBM were set as *L*_1_ = 16 mm, *α* = *β* = 30°, *t* = 0.4 mm, *t*_1_ = 2 mm, and *r* = 2 mm, with *θ* ranging from 20°, 30°, 40°, to 50°, and *t*_1_ varied at 1.2 mm, 1.4 mm, 1.6 mm, 1.8 mm, and 2.0 mm. The material used for the structure was Al–Invar. The FEA results in Figure 5g show *ν* transitions from positive to negative values as *t*_1_ increases between 1.6 mm and 1.8 mm, demonstrating that the positive and negative states of *ν* can be flexibly controlled by adjusting *t*_1_. Specifically, at *t*_1_ = 1.2 mm, larger *θ* values correspond to higher *ν* values. Conversely, at *t*_1_ = 1.4 mm, *ν* remains largely consistent across different *θ* angles. With further increases in *t*_1_, the NPR effect becomes more pronounced at larger *θ* values. Notably, the NPR effect is maximized at *θ* = 50° and *t*_1_ = 2.0 mm. The Poisson’s ratio (PR) exhibited a pronounced dependence on thickness. Specifically, a decrease of approximately 30% was observed as the thickness increased from 1.2 mm to 1.4 mm, while a more substantial reduction of approximately 150% occurred when the thickness increased from 1.2 mm to 2.0 mm. This nonlinear change suggests a transition in deformation mechanisms from bending-dominated to stretching-dominated behavior, enabling flexible and effective control of the mechanical response through thickness modulation.

A displacement constraint of 1 mm in the negative *x*-axis direction was imposed on the right boundary of the BTBM, while the left boundary was subjected to a displacement constraint of 1 mm in the positive *x*-axis direction. The upper and lower boundaries remained unconstrained. The resulting of the FEA is depicted in Figure 5h. As the angle *θ* increases, *ν* remains largely unchanged, contrasting with the observations made under longitudinal displacement constraints. This discrepancy suggests that the properties of the BTBM exhibit anisotropic characteristics.

### 3.2. Parameter Analysis of the Effective CTE

Due to the anisotropic nature of the BTBM’s properties, the transverse and longitudinal thermal expansion coefficients of the BTBM have been calculated separately for analysis and study. For enhanced convenience in result analysis, dimensionless parameters α_x_* and α_y_* are employed, where α* is defined as *α*/*α*_2_.

**Figure 6:**
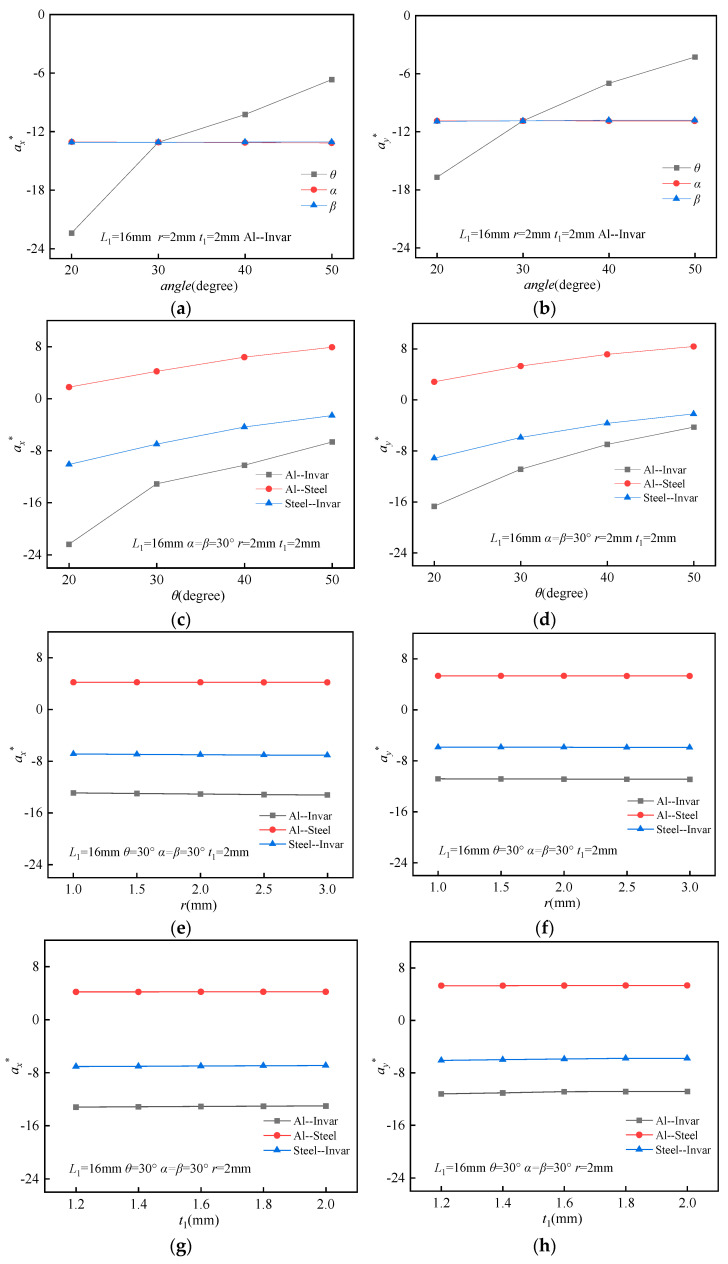
FEA of effective thermal expansion coefficients (*α*_x_* and *α*_y_*): (**a**) *θ*, *α*, *β* interactions on *α*_x_*; (**b**) *θ*, *α*, *β* interactions on *α*_y_*; (**c**) material combinations variation on *α*_x_*; (**d**) material combinations variation on *α*_y_*; (**e**) *r* variation on *α*_x_*; (**f**) *r* variation on *α*_y_*; (**g**) *t*_1_ variation on *α*_x_*; (**h**) *t*_1_ variation on *α*_y_*.

To systematically investigate the influence of the triangle angle (*θ*), thick beam angle (*α*), and thin beam angle (*β*) on the CTE, the geometric parameters of the BTBM were fixed as follows: *L*_1_ = 16 mm, *t* = 0.4 mm, *t*_1_ = 2 mm, and *r* = 2 mm. The material used for the structure was Al–Invar. The angles *θ*, *α*, and *β* were varied across four values: 20°, 30°, 40°, and 50°, respectively. The FEA results are presented in Figure 6a. It is observed that with increasing *θ*, the *α*_x_* and *α*_y_* values increase, while α and *β* have no significant influence on these parameters. This suggests that variations in *θ* play a dominant role in determining the CTE, whereas *α* and *β* remain largely independent of this behavior.

To investigate the impact of the angle *θ* on the CTE under varying material combinations, the size parameters of the BTBM were kept constant at *L*_1_ = 16 mm, *α* = *β* = 30°, *t* = 0.4 mm, *t*_1_ = 2 mm, and *r* = 2 mm, while the parameter *θ* was varied to 20°, 30°, 40°, and 50°, respectively. For the study, three distinct material combinations were selected: Al–Invar, Al–Steel, and Steel–Invar. The FEA results are presented in Figure 6b. As *θ* increases, the *α*_x_* and *α*_y_* for all three material combinations exhibit an upward trend, indicating the occurrence of the NTE effect. Furthermore, with the increasing *θ*, the deformation of the bi-material triangular foundation member in the height direction decreases, resulting in a weakening of the NTE effect. When *θ* remains constant, the Al–Invar material combination exhibits the smallest *α*_x_* and *α*_y_* values, while the Al–Steel composition possesses the largest *α*_x_* and *α*_y_* values.

To examine the effect of the ring radius (*r*) on the coefficient of the CTE under various material combinations, the following size parameters of the BTBM were chosen: *L*_1_ = 16 mm, *θ* = 30°, *α* = *β* = 30°, *t* = 0.4 mm, and *t*_1_ = 2.0 mm. The *r* was varied to 1.0 mm, 1.5 mm, 2.0 mm, 2.5 mm, and 3.0 mm. The FEA results are presented in Figure 6c. It is observed that with increasing *r*, the *α*_x_* and *α*_y_* for the three material combinations remain largely unchanged, suggesting that variations in *r* alone do not significantly influence the CTE.

To further investigate the influence of the wall thickness (*t*_1_) of the longitudinal unit of on the CTE under different material combinations, the following size parameters were selected: *L*_1_ = 16 mm, *θ* = 30°, *α* = *β* = 30°, *t* = 0.4 mm, and *r* = 2 mm. The thickness *t*_1_ was varied to 1.2 mm, 1.4 mm, 1.6 mm, 1.8 mm, and 2.0 mm. The FEA results are presented in Figure 6d. Similarly, with increasing *t*_1_, the *α*_x_* and *α*_y_* values for the three material combinations remain largely unchanged, indicating that variations in *t*_1_ alone do not significantly affect the CTE.

### 3.3. Parameter Analysis of Bandgap Properties

**Figure 7:**
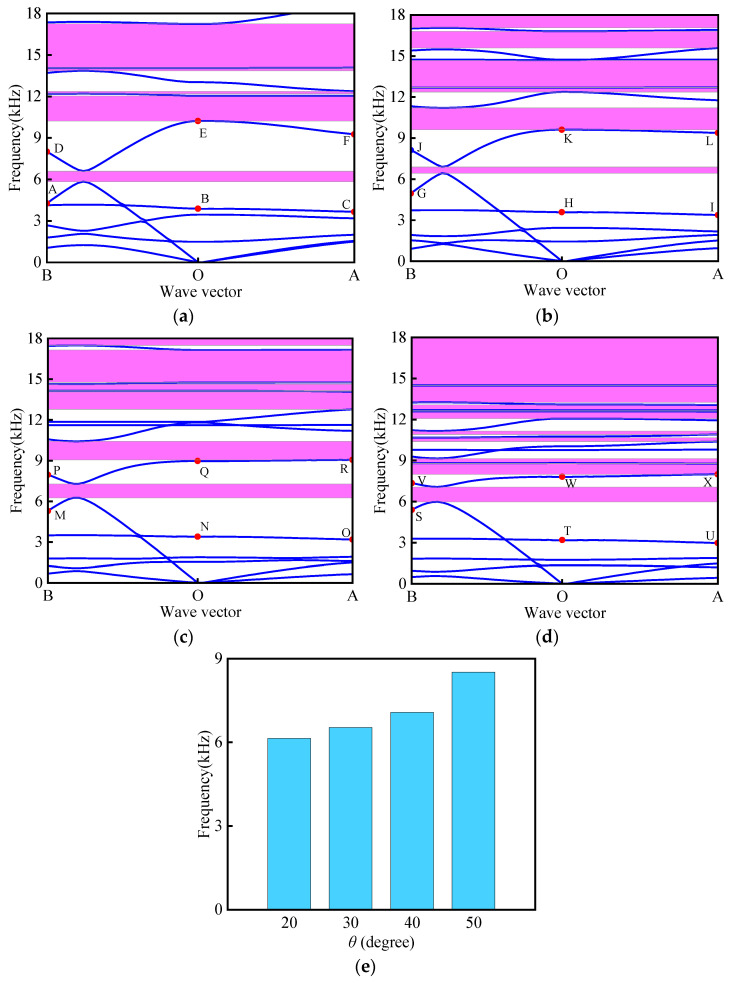
Influence of different angles *θ* on band structure: (**a**) *θ* = 20°; (**b**) *θ* = 30°; (**c**) *θ* = 40°; (**d**) *θ* = 50°; (**e**) total effective bandgap width.

A systematic investigation was conducted to evaluate the influence of angle *θ* variations on both the band structure characteristics and the total effective bandgap width. During the study, the size parameters of the BTBM were kept constant at *L*_1_ = 16 mm, *α* = *β* = 30°, *t* = 0.4 mm, *t*_1_ = 2 mm, and *r* = 2 mm, while *θ* was varied to 20°, 30°, 40°, and 50°, respectively. The material combination of Al–Invar was selected for the research. As depicted in Figure 7, at *θ* = 20°, five band gaps were observed, including a medium–high frequency band gap with a width of 764.8 Hz near 6000 Hz. At *θ* = 30°, six band gaps were generated, and the mid-high frequency band gap near 6000 Hz narrowed to 241.1 Hz. At *θ* = 40°, six band gaps were present, and with an increasing angle, the band gap width also increased. Finally, at *θ* = 50°, eleven band gaps were observed, with the middle and high-frequency bands gradually converging, while the high-frequency band gap broadened significantly, generating a high-frequency band gap with a width of 3482.8 Hz near 15,000 Hz. As shown in Figure 7e, the total effective bandgap width exhibits monotonic enhancement with increasing *θ*, peaking at 8513.6 Hz for *θ* = 50° (a 62% increase versus *θ* = 30°).

**Figure 8:**
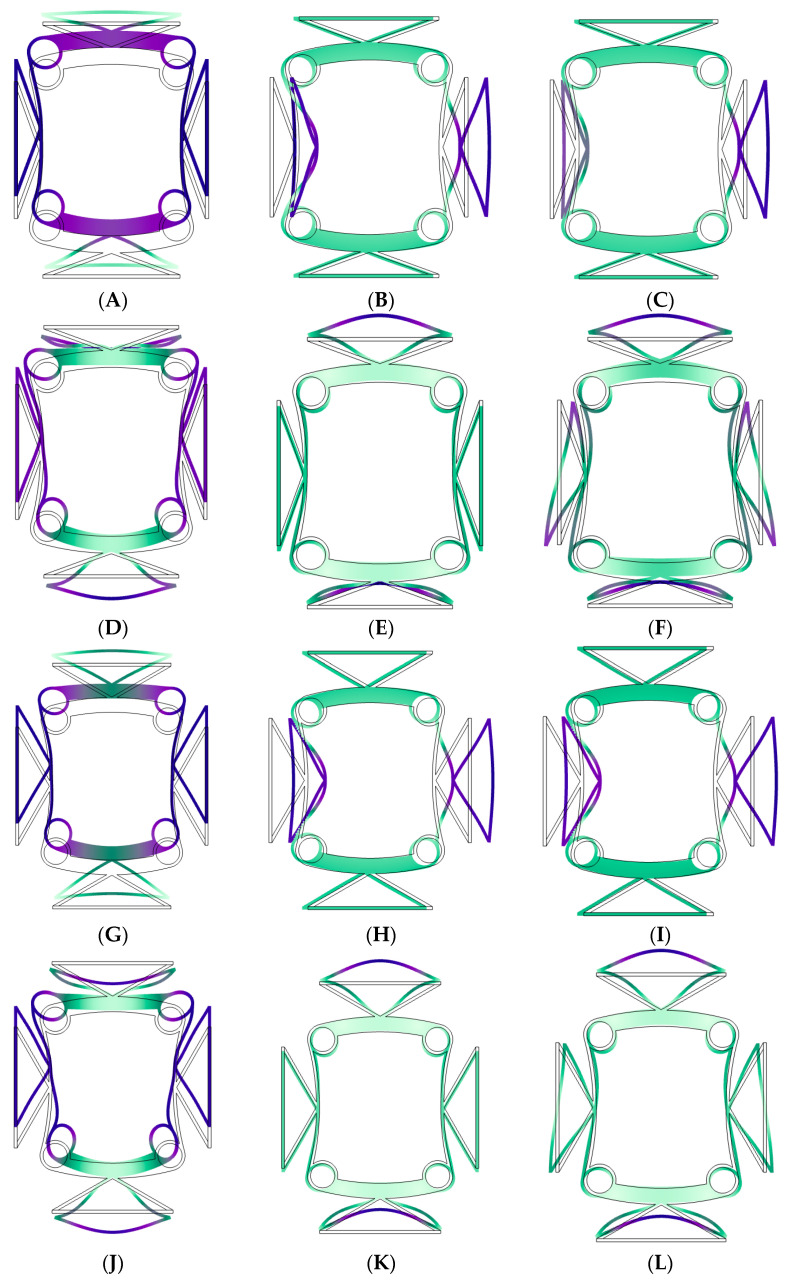

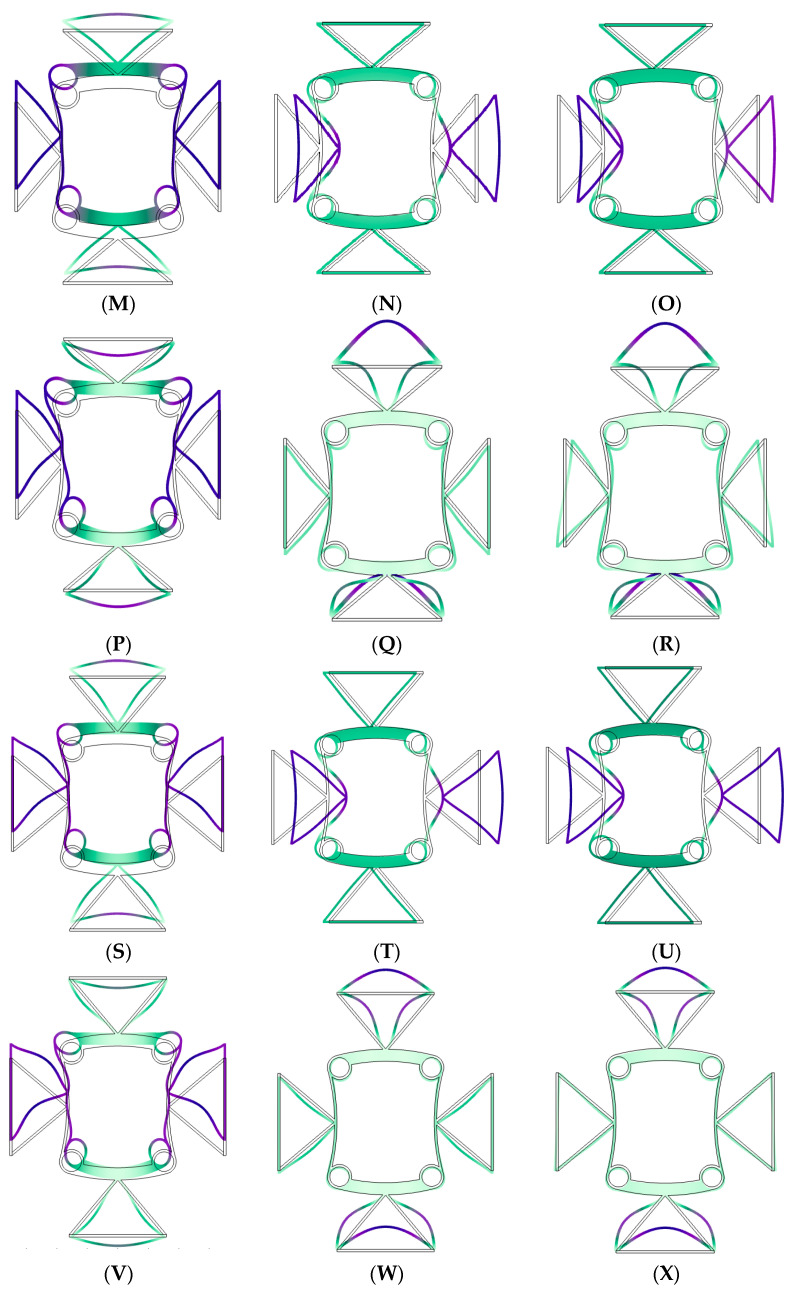
(**A**–**X**) points corresponding vibration modes.

To further elucidate the underlying formation mechanism of the band gap within the band structure, an analysis was conducted on the vibration modes at points B, O, and A, located at the upper and lower boundaries of the first band gap. As illustrated in Figure 8, the vibration modes at points A, G, M, and S are characterized by upward tensile deformation of the upper bi-material triangle, upward compressive deformation of the lower bi-material triangle, inward compressive deformation of the left and right bi-material triangles, and an overall upward movement of the structure. The vibration modes observed at points B, C, H, I, N, O, T, and U are dominated by the right compression curved beam deformation of the left bi-material triangle and the right extension curved beam deformation of the right bi-material triangle. The vibration modes at points D, J, P, and V are mainly manifested as downward compression deformation of the upper bi-material triangle and downward tensile deformation of the lower bi-material triangle. The analysis further revealed that the vibration modes at points E, K, Q, and W are primarily characterized by upward tensile deformation of the upper bi-material triangle and upward compression deformation of the lower bi-material triangle. Additionally, at points F, L, R, and X, the vibration modes exhibit not only the aforementioned tensile and compressive deformations but also rotational deformation of the left and right triangles.

**Figure 9:**
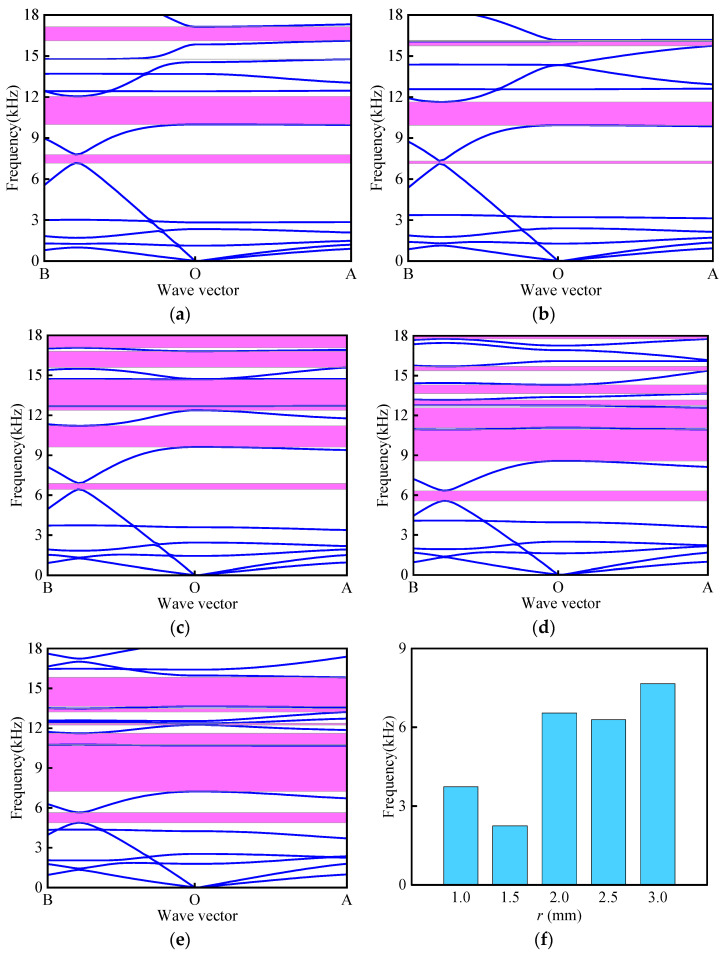
Influence of ring radius *r* of curved beam on band structure: (**a**) *r* = 1 mm; (**b**) *r* = 1.5 mm; (**c**) *r* = 2 mm; (**d**) *r* = 2.5 mm; (**e**) *r* = 3 mm; (**f**) total effective bandgap width.

To investigate the impact of the ring radius (*r*) on the band structure and the total effective band gap width, the size parameters of the BTBM were fixed at *L*_1_ = 16 mm, *θ* = 30°, *α* = *β* = 30°, *t* = 0.4 mm, and *t*_1_ = 2 mm, respectively. The material combination of Al–Invar was selected, and the radius *r* was varied to 1.0 mm, 1.5 mm, 2.0 mm, 2.5 mm, and 3.0 mm. As depicted in Figure 9, as *r* increases from 1 mm to 1.5 mm, four band gaps are observed, with all band gaps progressively narrowing and the band gap near 15,000 Hz disappearing completely. When *r* is increased to 2 mm, six band gaps are generated, with the mid-to-high-frequency band gap near 7000 Hz widening significantly, while multiple mid-to-high-frequency band gaps emerge above 12,000 Hz. At *r* = 2.5 mm, seven more dispersed band gaps are observed. Finally, when *r* reaches 3 mm, six band gaps are generated, with the band gap near 9000 Hz exhibiting significant broadening. The effect of *r* on the total effective band gap width is presented in Figure 9f. As the radius *r* increases from 1 mm to 1.5 mm, the total effective band gap width decreases. When *r* increases from 2 mm to 2.5 mm, although the band gap width shows a slight decrease, the overall trend remains increasing. Specifically, the minimum total effective band gap width of 2251.7 Hz occurs at *r* = 1.5 mm, while the maximum width reaches 7657.1 Hz when *r* = 3 mm.

**Figure 10:**
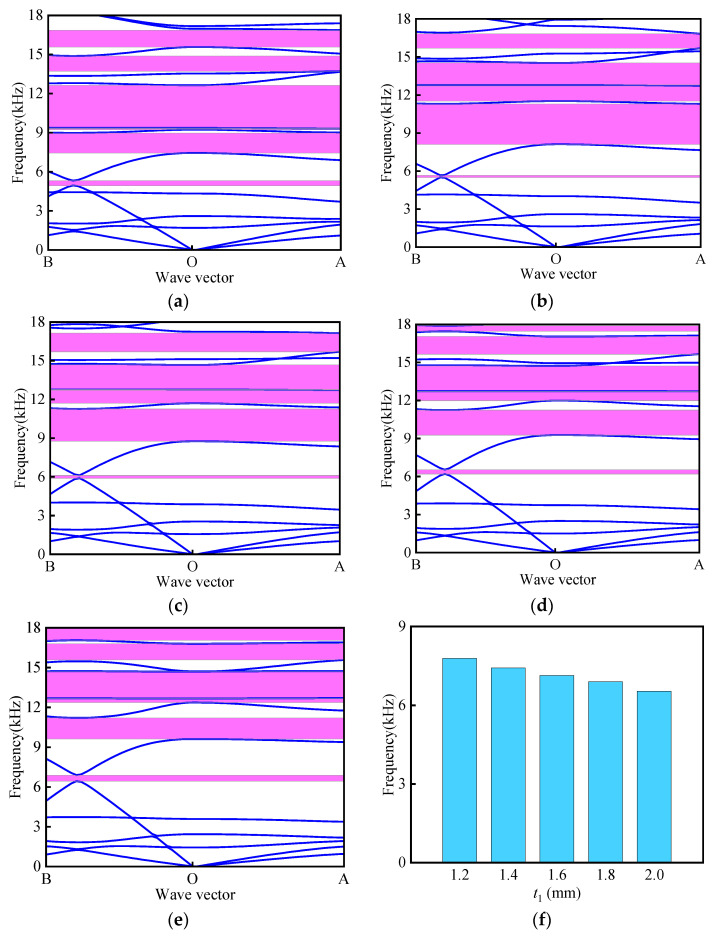
Influence of longitudinal unit wall thickness *t*_1_ on band structure of curved beam: (**a**) *t*_1_ = 1.2 mm; (**b**) *t*_1_ = 1.4 mm; (**c**) *t*_1_ = 1.6 mm; (**d**) *t*_1_ = 1.8 mm; (**e**) *t*_1_ = 2 mm; (**f**) total effective bandgap width.

To further investigate the influence of the wall thickness (*t*_1_) of the longitudinal unit on the band structure and the total effective band gap width under different material combinations, the following size parameters were selected: *L*_1_ = 16 mm, *θ* = 30°, *α* = *β* = 30°, *t* = 0.4 mm, and *r* = 2 mm. The thickness *t*_1_ was varied to 1.2 mm, 1.4 mm, 1.6 mm, 1.8 mm, and 2.0 mm. As illustrated in Figure 10, five band gaps are observed when *t*_1_ is set to 1.2 mm, 1.4 mm, and 1.6 mm, while six band gaps appear at *t*_1_ = 1.8 mm and 2.0 mm. With increasing *t*_1_, the overall variation trend in both the number and width of band gaps remains relatively stable. The impact of *t*_1_ on the total effective band gap width is depicted in Figure 10f, showing a decreasing trend as *t*_1_ increases. Specifically, the maximum total effective band gap width of 7788.5 Hz occurs at *t*_1_ = 1.2 mm, while the minimum width reaches 6533.6 Hz when *t*_1_ = 2.0 mm.

**Figure 11:**
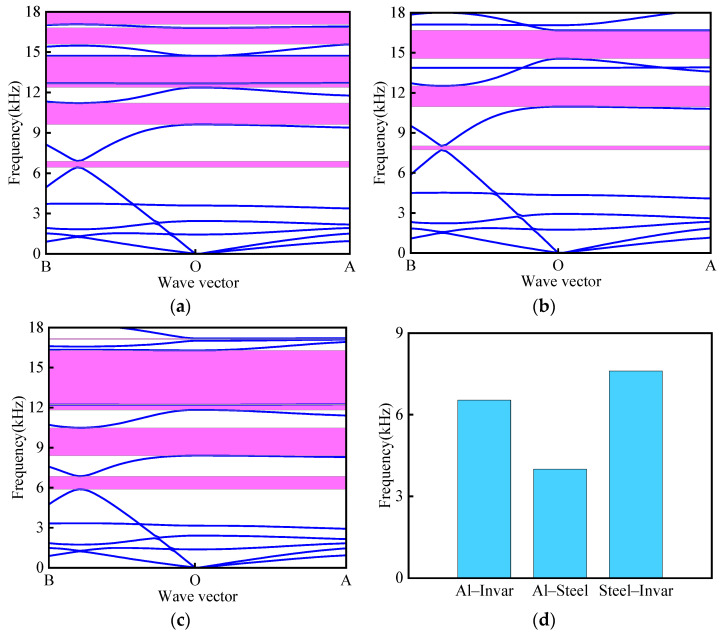
Influence of different material combinations on band structure: (**a**) Al– Invar; (**b**) Al–Steel; (**c**) Steel–Invar; (**d**) total effective bandgap width.

To investigate the impact of various material combinations on the band structure and the total effective band gap width, the following size parameters were selected: *L*_1_ = 16 mm, *θ* = 30°, *α* = *β* = 30°, *t* = 0.4 mm, *t*_1_ = 2 mm, and *r* = 2 mm. Three distinct material combinations were studied: Al–Invar, Al–Steel, and Steel–Invar. As depicted in Figure 11, the Al–Invar material combination generates six band gaps, while the Al–Steel combination produces three band gaps and the Steel–Invar combination yields five band gaps. Notably, the Stee–-Invar combination creates a significantly wider mid-to-high-frequency band gap near 15,000 Hz. As evidenced in Figure 11d, the choice of material combinations critically influences the total effective bandgap width, with variations exceeding 40% between different pairings. Among the tested combinations, the Al–Steel combination exhibits the minimum total effective band gap width of 3994.2 Hz, while the Steel–Invar combination achieves the maximum total effective band gap width of 7603.7 Hz.

## 4. Conclusions

In this study, a bi-material triangle curved beam honeycomb metamaterial exhibiting tunable Poisson’s ratio, thermal expansion, and band gap characteristics has been successfully designed. Utilizing the Bloch theorem and finite element simulation analysis, the energy band structure was calculated, and its accuracy was verified through the analysis of transmission characteristic curves. A comprehensive parametric study was conducted to evaluate the effects of key geometric variables (*θ*, *r*, *t*_1_) and material systems (Al–Invar, Al–Steel, Steel–Invar) on the structural mechanical performance. By adjusting the geometric parameters and material combinations, four distinct types of metamaterials can be achieved: positive thermal expansion combined with negative Poisson’s ratio (PTE+NPR), zero thermal expansion combined with positive Poisson’s ratio (ZTE+PPR), negative thermal expansion combined with positive Poisson’s ratio (NTE+PPR), and negative thermal expansion combined with negative Poisson’s ratio (NTE+NPR). The proposed metamaterial exhibits a broader tunable range of Poisson’s ratio compared to existing designs. Notably, all four types of metamaterials demonstrate band gap characteristics, thereby exhibiting excellent sound insulation performance. This research demonstrates that through the rational selection of geometric parameters and material combinations, the coupling adjustment of CTE and PR can be achieved. Additionally, a significant total effective band gap width can be attained, enabling the realization of multifunctional integration. Compared to the current state-of-the-art metamaterial designs, which typically focus on tuning either Poisson’s ratio or thermal expansion independently, the proposed BTBM structure simultaneously achieves broad tunability in both mechanical and acoustic properties within a single integrated framework. This level of multifunctional integration represents a substantial advancement over previously reported works, highlighting the uniqueness and versatility of the proposed design. This innovative design holds immense potential for applications in various fields, including vibration isolation, noise reduction, and thermal management, where the tailored properties of these metamaterials can be harnessed to optimize performance.

## Figures and Tables

**Table 1:** Material properties of Al, Steel, and Invar.

Material	Young’s Modulus *E* (GPa)	Poisson’s Ratio *v*	Density ρ (kg/m^3^)	CTE α (ppm/°C)
Al	71	0.3	2810	24.0
Steel	206	0.3	7800	13.0
Invar	144	0.29	8050	1.2

## Data Availability

The original contributions presented in this study are included in the article. Further inquiries can be directed at the corresponding authors.
